# Dynamic Path Planning of AGV Based on Kinematical Constraint A* Algorithm and Following DWA Fusion Algorithms

**DOI:** 10.3390/s23084102

**Published:** 2023-04-19

**Authors:** Xiong Yin, Ping Cai, Kangwen Zhao, Yu Zhang, Qian Zhou, Daojin Yao

**Affiliations:** School of Electrical and Automation Engineering, East China Jiaotong University, Nanchang 330000, China

**Keywords:** A* algorithm, DWA algorithm, global path planning, dynamic obstacle avoidance

## Abstract

In the field of AGV, a path planning algorithm is always a heated area. However, traditional path planning algorithms have many disadvantages. To solve these problems, this paper proposes a fusion algorithm that combines the kinematical constraint A* algorithm and the following dynamic window approach algorithm. The kinematical constraint A* algorithm can plan the global path. Firstly, the node optimization can reduce the number of child nodes. Secondly, improving the heuristic function can increase efficiency of path planning. Thirdly, the secondary redundancy can reduce the number of redundant nodes. Finally, the B spline curve can make the global path conform to the dynamic characteristics of AGV. The following DWA algorithm can be dynamic path planning and allow the AGV to avoidance moving obstacle. The optimization heuristic function of the local path is closer to the global optimal path. The simulation results show that, compared with the fusion algorithm of traditional A* algorithm and traditional DWA algorithm, the fusion algorithm reduces the length of path by 3.6%, time of path by 6.7% and the number of turns of final path by 25%.

## 1. Introduction

In recent years, due to the great market demands, broad application prospects and large potential development, mobile robots are widely used in transportation systems, e.g., for inspecting high-speed railway stations, sweeping roads and intelligently managing traffic [1,2]. Mobile robots are widely used in the coal mining industry, e.g., as a mining robot, coal robot and so on. Intelligent mining machinery can improve the scientific nature and efficiency of mining [3,4]. Mobile robots are widely used in the military field, e.g., as reconnaissance robots, supplies robots, attack robots and so on [5]. Intelligent military equipment can improve the combat capability and level of troops [6]. With the development of science, technology and the innovation of automobile technology [7], autonomous vehicles have gradually developed into intelligent networked vehicles, which continuously improve traffic safety and efficiency. Path planning is also an important bridge between the environment awareness module and the tracking control module of the autonomous vehicle [8]. With the modern agricultural production process moving towards intelligence, information, scale and refinement, agricultural machinery path planning technology is one of the basic technologies of intelligent agricultural equipment [9]. It not only can improve the quality of agricultural machinery operation, but also can make agricultural production standardization and normalization [10]. It promotes the efficient production of smart agriculture [11].

It is worth pointing out that most of the aforementioned path planning algorithms have a number of disadvantages, such as low search efficiency, discontinuous curvature and long path. Luo Q [12] shows that the unequal allocation of initial pheromones is constructed. The pseudo random states transition rule is used for route selection. Moreover, the proportion of determined or random selection is adjusted adaptively. Sang H [13] has shown that the optimal path is divided into multiple sub-target points to form a sub-punctuation sequence. The UAV is removed from the local minimum by switching target points. Tang G [14] suggests that the functions P (x, y) and W (x, y) are used to filter the nodes in the close list. Remove the nodes that do not meet the requirements; replace the broken lines on the turning path to cubic B-spline curves. Wang H [15] recommends extended distance, two-way search and smoothing into path planning for optimizing path. Bayerlein H [16] proposes a multi-agent reinforcement learning which can adapt to profound changes in the scenario parameters defining the data harvesting mission. The path planning problem is also transformed into a decentralized partially observable Markovian decision process, using deep reinforcement learning methods to solve. Zhou Y [17] recommends that the predicted and observed values be modified by Kalman filter gain coefficient to obtain the final target tracking results. This reduces the average error and makes the trajectory closer to the real trajectory of vehicle.

Many studies have focused on finding the optimized path to carry out the task [18]. This is divided into two parts in the path planning process of a mobile robot from the starting position of the target position: one is the global path planning, the other one is the local path planning [19]. The common global path planning algorithms have the following disadvantages: (1) The path contains a large number of redundant nodes [20]; (2) The number of path turns is large [21]; (3) The path does not conform to the kinematic characteristics of the AGV. The local path planning algorithms have the following disadvantages [22]: (1) The path tends to fall into local optimality; (2) The path may not be found [23]; (3) AGV cannot avoid dynamic obstacle [24].

The traditional path planning method has the disadvantages mentioned above, so this paper proposes a fusion algorithm that combines the kinematical constraint A* algorithm and the following dynamic window approach. The global path is shorter and smoother. The local path is closer to the global path and can avoid dynamic obstacle [25].

In summary, the fusion algorithm is proposed to achieve a mobile robot that can both travel through a globally optimal path and avoid local dynamic obstacle. Compared with the existing work, we make three contributions:

(1) Improve the child node selection method and heuristic function of A* algorithm. This can improve the search efficiency of the algorithm. Reduce computing burden. Make the generated path more realistic;
(2) For secondary redundant node removal, use B spline curve for smoothness constraint. This reduces the number of redundant points in the path. The generated path conforms to the dynamic constraints;
(3) Intercept the key node information and apply improved DWA algorithm to make the local path planning follow the global path contour, so as to make the path smoother and achieve avoidance of local dynamic obstacle. It can prevent DWA algorithm from falling into local optimal. It allows the mobile robot to avoid moving obstacles.

The rest of this paper is organized as follows. Section 2 describes the disadvantages of traditional A* and improves the A* algorithm. Section 3 presents the improvement of DWA algorithm, the improved A* algorithm and the improved DWA algorithm fusion. Section 4 introduces the algorithm for simulation experiment and analysis of experimental values. Finally, Section 5 concludes our work and describes some future research issues.

## 2. Improved A* Algorithm

In the grid map, there is a heuristic search strategy based on the evaluation function of the traditional A* algorithm, which can find a long obstacle-free path of a static environment. However, there are a number of turn points and redundant points in this unsmooth path, which is unconformity kinematic. The car may not be able to go along this path, which is a tangent to the apex of obstacles. In this paper, we improve the traditional A* according to the motion characteristics and map information.

### 2.1. Node Optimization

**Figure 1:**
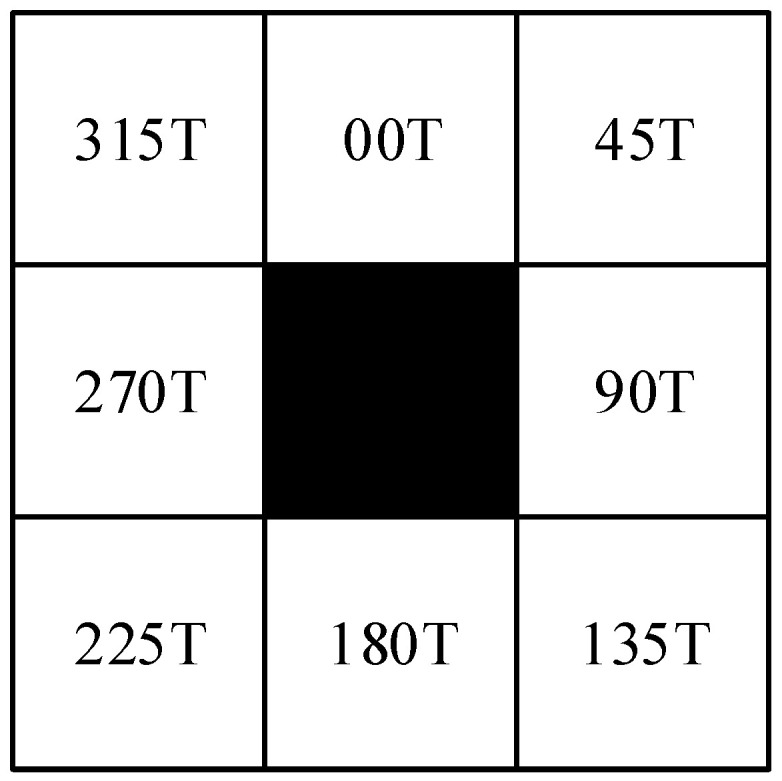
Node search direction.

In the mobile robot industry, the traditional A* algorithm has long been used for planning purposes, but there is still the difficulty of avoiding moving obstacle and temporary obstacle. The influence of node search strategy has shown a lot of redundant points and infection points. The traditional A* algorithm search principle is to add the starting point in the open list. Then, add the point as the father node to the close list. Next, search its neighboring children’s nodes in 8 directions [3]. It visits nodes in a graph from the start node to the goal node. However, its simple search tactic is rigid when oriented to different environments. In most cases, the obstacle environment in which the mobile robot is located is not complex. The number of search directions can be reduced at this time. In this paper, we propose a five sub-nodes search method, which connects the current node with the target node and sets its angle with Y circumference as θ. θ can be obtained from Equation (1). With the node as the center, there is no obstacle to this region and the search direction will be reduced from 8 to 5, as shown in the Figure 1 and the search direction from Table 1.

(1) $$\theta =\arctan\frac{g_{y}-n_{y}}{g_{x}-n_{x}}$$

### 2.2. Improved Heuristic Function

A* algorithm is a graph search algorithm depended on the heuristic selection of the heuristic function [26]. Heuristic information related to the characteristics of the problem is utilized to guide its performance. Calculate the cost of the nodes in the open list based on the evaluation function. Select the node with the lowest cost as the next parent node. Put it into the close list. Search the parent reachable node again. Calculate its cost and so on until the parent node is the location of the target point. It is easy to see that $h\left(n\right)$ has a great impact on the search efficiency:

(2) $$h(n)=[{\left(n_{x}-g_{x}\right)}^{2}+{\left(n_{y}-g_{y}\right)}^{2}]$$

This is because the heuristic function is Euclidean distance. The estimated value is smaller than the actual surrogate value. This paper proposes increase the ratio of $h\left(n\right)$ in $f\left(n\right)$. The basic form of the evaluation function is as follows:

(3) f(n)=g(n)+h(n)

The optimized form of the evaluation function is as follows:

(4) f(n)=g(n)+(1.5+r/R)∗h(n)

where $g\left(n\right)$ is the actual cost from the starting point of the AGV path to the current node, r is the distance from the current point to the target point and R is the distance from the start point to the target point.

### 2.3. Redundancy Removal

The secondary removal of redundant points is improved on the basis of the ordinary removal of redundant points [27]. The algorithm of common redundancy removal takes the greedy algorithm as an example. The greedy algorithm selects the optimal parent node in the current condition. The parent node it selects is added with collision detection function. The final generated path will not collide with the obstacle. Connect the target point and the initial point in collision detection. If the collision means that it cannot be omitted, then the target node’s previous father node is set as the target point to repeat the above process. If no collision occurs, this means that it can be omitted. The detection ends when the father node is the starting point. Moreover, the path point that does not collide will generate the de-redundant trajectory.

**Figure 2:**
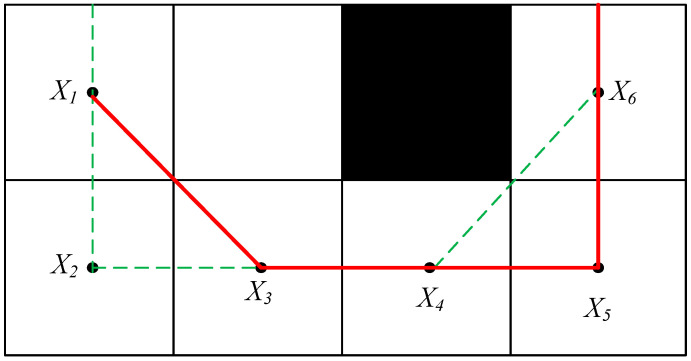
Secondary de-redundant path.

However, the normal redundancy removal is not sufficient and the path generated by it will be tangent to the vertices of the obstacles. In this case, the generated path is apparently collision-free. When the mobile robot travels along the path, it will collide with the obstacle when it reaches the vertex of the obstacle. Moreover, no space is reserved for the smoothness constraint on the subsequent path. Therefore, the common algorithm of removing redundant points is insufficient. This paper proposes a new algorithm—a redundant point secondary removal algorithm—when the distance between all nodes and the center point of the obstacle is greater than the preset safety distance. Then, the nodes between the two points that form a straight line are considered redundant nodes and can be removed. This can happen when there is any equilibrium point and the distance between the center point of the obstacle is less than the preset safety distance. The redundancy cannot be pointed removal operation. In this paper, the safety distance is the length of the edge of a grid.

(5) $$k=ceil(norm(M_{i+2}-M_{i})*k_{1})$$

where norm() function is used to find the Euclidean norm, and the ceil() function is used to find the nearest integer that is not smaller than the number in parentheses, with $\;k_{1}$ being any positive integer. As shown in Figure 2, the unremoved redundant path is X_1_ → X_2_ → X_3_ → X_4_ → X_6_. If removing this point can shorten the length of the path, it is not tangent to the vertex of the obstacle. It is regarded as a point that can be removed and the points on the path are judged. Finally, the redundancy removal path can be obtained X_1_ → X_3_ → X_4_ → X_5_ → X_6_.

### 2.4. B Spline Curve Smoothing Constraints

Considering the kinematic characteristics of the mobile robot [28,29], it is necessary to constrain the smoothness of the path after the redundancy has been removed by a B spline curve, which can generate a smooth path to continuous curvature by using the path points put into the path after the redundancy has been removed by the greedy algorithm as the control points of the B spline curve basis function. This can be achieved by using a B spline curve to constrain the smoothness of the path after redundancy removal [30,31]. Not only can the order be specified, but also changing the control points will change the shape of part of the curve, which retains the advantages of the Bezier curve and optimizes the shortage of the Bezier curve that cannot be modified in the local path.

Let there exist n+1 control points $P_{i}\left(i=0,1,2,\ldots ,n\right)$, node vector $T=\left[t_{0},\;t_{1},\ldots ,\;t_{n+k+1}\right]$ and k-order B spline curve can be defined as follows.

(6) $$P(t)=\sum_{i=0}^{n}P_{i}B_{i,k}(t)$$

(7) $$B_{i,k}(t)=\frac{1}{k!}\sum_{j=0}^{k-1}{\left(-1\right)}^{j}C_{k+1}^{j}{\left(t+k-i-j\right)}^{k}$$

where $P\left(t\right)$ is B-spline curve function and $B_{i,k}\left(t\right)$ is k-order B-spline basis function.

The paths with redundant points removed are smoothed using the cubic B spline curve constraint. Moreover, the cubic B spline curve equation are (A1)–(A3).

**Figure 3:**
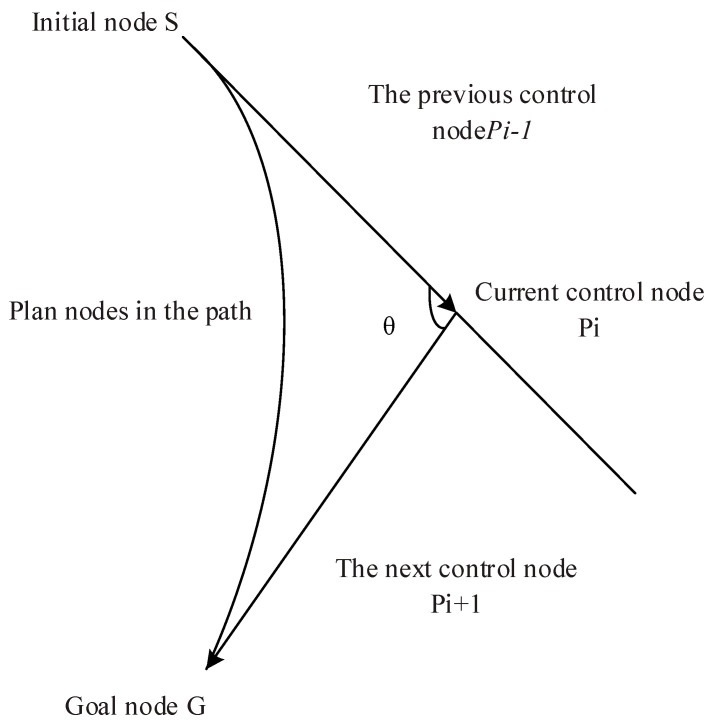
B spline curve theory.

This is how the equation can be expressed in matrix form:

(8) $$P(t)=\frac{1}{6}[\begin{array}{cccc} 1 & t^{} & t^{2} & t^{3} \end{array}]\left[\begin{array}{cccc} 1 & 4 & 1 & 0\\ -3 & 0 & 3 & 0\\ 3 & -6 & 3 & 0\\ -1 & 3 & 3 & 1 \end{array}\right]\left[\begin{array}{c} P_{0}\\ P_{1}\\ P_{2}\\ P_{3} \end{array}\right]$$

where $P_{0}$, $P_{1}$, $P_{2}$, $P_{3}$ are control points, as shown in Figure 3.

**Figure 4:**
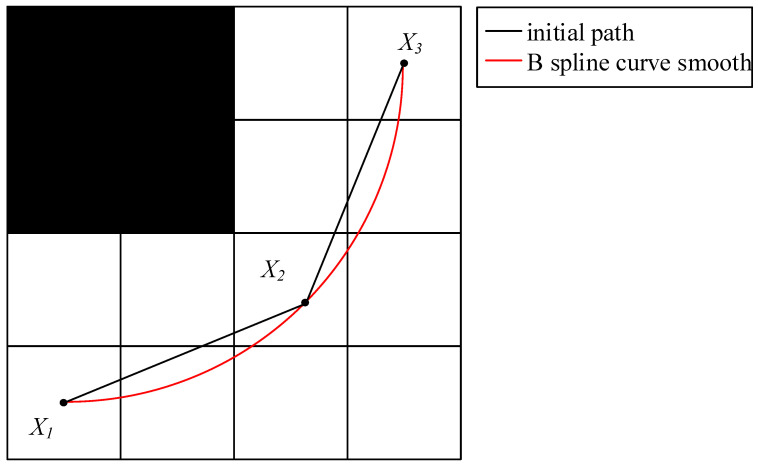
B spline curve diagram.

In this paper, a B spline curve is used to smoothly fit the path after removing redundancy, and the smoothed path is shown in Figure 4.

## 3. Improved DWA Algorithm

Dynamic window algorithm (DWA algorithm) is a common algorithm for solving local obstacle avoidance of mobile robots. The algorithm is designed to sample multiple sets of velocities in the velocity space based on the motion model of the mobile robot. Analyze and predict the trajectory of the mobile robot at each set of velocities over a period of time. Then, select the velocity corresponding to the optimal trajectory according to the evaluation function of the algorithm. Drive mobile robot for local path planning according to velocity corresponding to the optimal trajectory.

### 3.1. Motion Model of Mobile Robot

The DWA algorithm samples the linear and angular velocities of the mobile robot in the window region, so the first step requires kinematic modeling of the mobile robot. Assume that the mobile robot moves in a uniform linear motion for a period of time $\left(\Delta t\right)$. Denote the kinematic model of the mobile robot during that time period.

(9) $$\left\{\begin{array}{c} x(t)=x(t-1)+\upsilon (t)\Delta t\cos(\theta_{t-1})\\ y(t)=y(t-1)+\upsilon (t)\Delta t\sin(\theta_{t-1})\\ \theta_{t}=\theta_{t-1}+\omega (t)\Delta t \end{array}\right.$$

### 3.2. Speed Sampling for Mobile Robots

In the mobile robot velocity group space, there are theoretically infinite sets of velocity sets $\left(\upsilon ,\;\omega \right)$. However, the mobile robot is easily constrained by its own hardware and working environment. It is necessary to constrain the range of sampled velocity sets according to the actual situation after obtaining the robot motion model.

The maximum and minimum linear velocity $\upsilon_{max}$, $\upsilon_{min}$ and angular velocity $\omega_{max}$, $\omega_{min}$ ranges of a mobile robot constrained by its own conditions can be expressed as:

(10) $$V_{m}=\{\left(\upsilon ,\omega )|\upsilon \in \left[\upsilon_{min},\upsilon_{max}\right]\cap \omega \in \left[\omega_{min},\omega_{max}\right]\right\}$$

The mobile robot is affected by its own motor. There is a deceleration constraint. Within the dynamic window display, the mobile robot is affected by the acceleration of the maximum. The minimum speed range is:

(11) $$V_{d}=\left\{\begin{array}{c} \left(\upsilon ,\omega )|\upsilon \in [\upsilon_{t}-\upsilon_{a}\Delta t,\upsilon_{t}+\upsilon_{a}\Delta t\right]\\ \omega \in [\omega_{t}-\omega_{a}\Delta t,\omega_{t}+\omega_{a}\Delta t] \end{array}\right\}$$

where $\upsilon_{t}$ is the current linear velocity, $\upsilon_{a}$ is linear acceleration, $\omega_{t}$ is the current angular velocity and $\;\omega_{a}$ is angular acceleration.

Mobile robot braking distance constraint. The realization of dynamic obstacle avoidance depends mainly on the braking distance constraint. The DWA algorithm looks for obstacles in the process of selecting the speed and trajectory evaluation. To ensure the reliability and safety of the mobile robot when it works, it needs its speed to be reduced to 0 under the maximum deceleration condition before colliding with the obstacle. The constraint Equation (12) is shown.

(12) $$V_{a}=\left\{\begin{array}{c} \left(\upsilon ,\omega )|\upsilon \leq sqrt[2dist(\upsilon ,\omega )\cdot \upsilon_{a}\right]\\ \omega \leq sqrt[2dist(\upsilon ,\omega )\cdot \omega_{a}] \end{array}\right\}$$

### 3.3. Optimization of Evaluation Function

The evaluation function in the DWA algorithm is used to select the optimal trajectory. The criteria for the regulation evaluation are: accurate avoidance of obstacles and the shortest time-consuming approach to the target point. The design evaluation function is:

(13) G(υ,ω)=σ[αhead(υ,ω)+βdist(υ,ω)+γvel(υ,ω)]

where $head\left(\upsilon ,\;\omega \right)$, $dist\left(\upsilon ,\;\omega \right)$ and $vel\left(\upsilon ,\;\omega \right)$ are the azimuth, distance, velocity evaluation sub-functions, respectively. σ is the smoothing function. α, β, γ are the weighting coefficients of each evaluation sub-function. However, the target points of path of DWA algorithm are proposed by the global path. This paper optimizes the evaluation function that change the angle difference between the end direction of the trajectory and the final target point to the angle difference between the current target point:

(14) G(υ,ω)=σ[αPhead(υ,ω)+βdist(υ,ω)+γvel(υ,ω)]

where $PHead\left(\upsilon ,\;\omega \right)$ is the angular difference between the end direction of the trajectory and the current target point.

### 3.4. Algorithm Fusion

The improved A* algorithm obtains a smooth navigation path. However, if there was an obstacle to this route, the route would not be changed. The DWA algorithm has good local dynamic obstacle avoidance capability. However, it is easy to fall into local optimum or even no solution. Therefore, this algorithm fuses the improved A* algorithm with the DWA algorithm. Extract the key nodes in the global path generated by the improved A* algorithm. Use them as temporary target points of the DWA algorithm so that the local path planning follows the contour of the global path planning. By combining the two, the mobile robot is able to avoid dynamic obstacles while not colliding with static obstacles in the path planning process.

**Figure 5:**
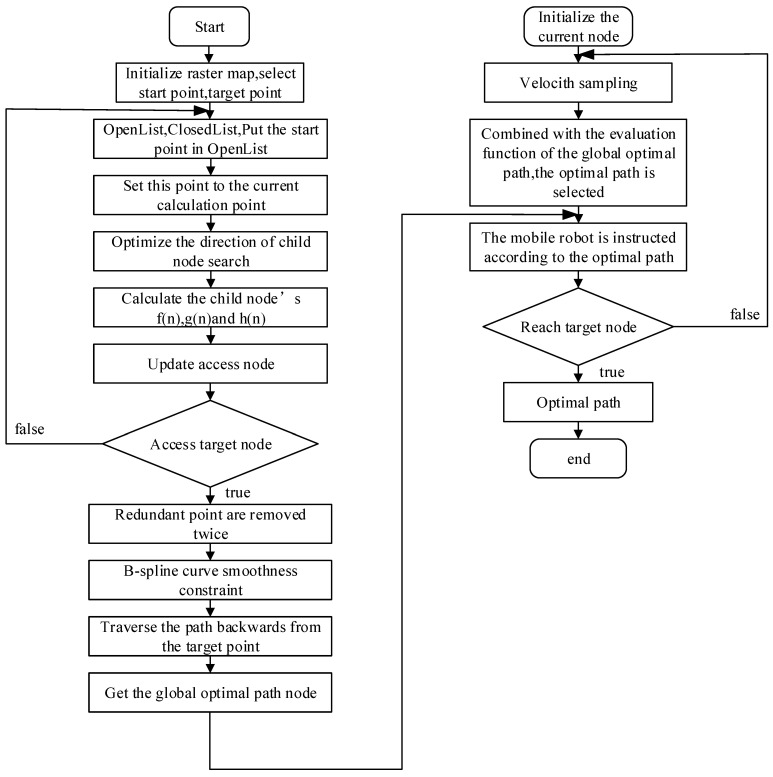
Fusion algorithm flow chart.

First, initialize the raster map, select the starting point and target point and generate an initial path using the improved A* algorithm. As the path may slice the obstacle fixed point, paths are de-redundant twice. Then, use the B spline as a smoothness constraint to obtain the global path. Extract the key nodes in the path. Thirdly, DWA algorithm receives the extracted key nodes as each target point of the DWA algorithm. The DWA algorithm kinematically models the mobile robot, samples the velocity and simulates the moving trajectory of each velocity. Fourthly, combine the evaluation function to select the optimal simulated moving trajectory. Move along the globally planned path with the optimal trajectory, while avoiding local dynamic obstacles. Finally, reach the target point. The flow chart of fusion algorithm is shown in Figure 5.

## 4. Experimental and Results

In order to test the feasibility and effectiveness of the improved algorithm in this paper, the running environment used is: MATLAB R2022a; Windows 10 64bit; processor (Intel) i5-12400F 12th generation; memory 16GB.

**Figure 6:**
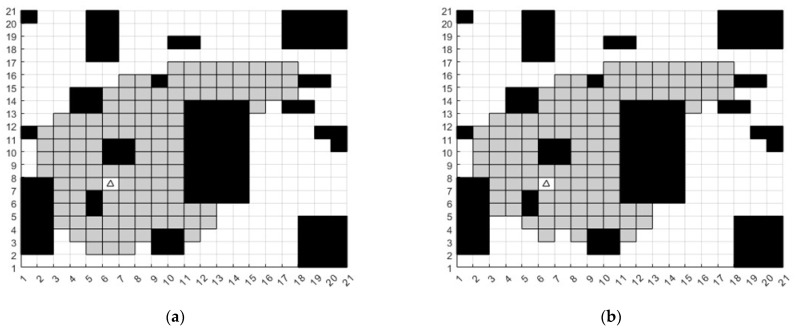
(**a**) 8-search direction simulation, (**b**) 5-search direction simulation.

In order to verify the validity of the improved child nodes search strategy, set the starting coordinates to (6, 7) and the ending coordinates to (17, 16). Then, conduct 8-direction search and 5-direction search, respectively, to obtain the 8-direction search node schematic and 5-direction search node schematic, which can obviously find the search node reduction. In the map, the black squares are obstacles. The triangle is the starting point, the circle is the goal point. The gray grid is the child node of the search. The black dotted line is the path. The number of children in 5-search direction decreased by 7.6% compared to the number in 8-search direction, as shown in Figure 6. In the figure, the black squares are obstacles. The gray squares are the children of the search. The triangle with coordinates (6, 7) is the starting point. The coordinate (17, 16) is the end point.

**Figure 7:**
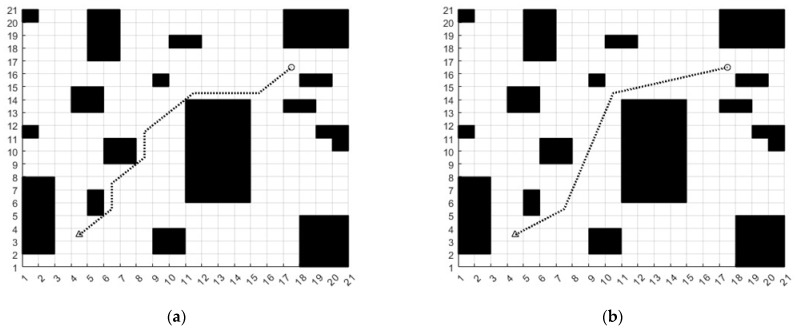
(**a**) Traditional A* path, (**b**) Secondary de-redundant path.

In order to verify the effectiveness of secondary de-redundancy, set the starting coordinates to (4, 3) and the ending coordinates to (17, 16). Then, a path with the redundancy points not removed and a path with the redundancy points removed are generated, respectively, which can obviously find that the number of turns is less. The length of path is shorter and the path is not tangent to the vertex of the obstacle. The number of turns in a path with the redundancy points removed decreased by 66.7%, as shown in Figure 7. In the figure, the black squares are obstacles. The black dotted line is the path. The triangle with coordinates (4, 3) is the starting point. The coordinate (17, 16) is the end point.

**Figure 8:**
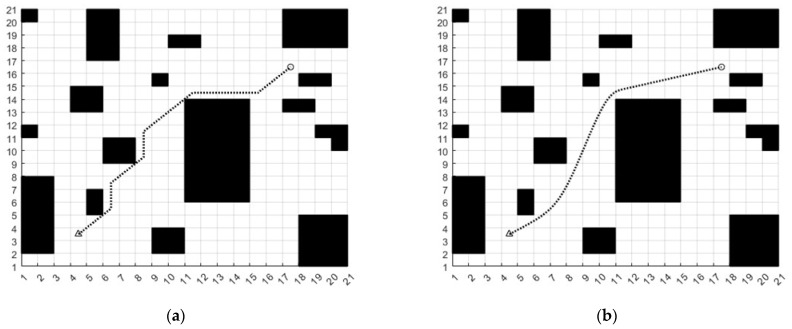
(**a**) Traditional A* path, (**b**) B spline smooth path.

In order to verify the effectiveness of the B spline curve, set the starting coordinates to (4, 3) and the ending coordinates to (17, 16). Then, an unsmoothed path and a smoothed B spline path are generated, respectively, which can obviously find that the smooth path of the B spline curve conforms to the kinematic characteristics of AGV, as shown in Figure 8. In the figure, the black squares are obstacles. The black dotted line is the path. The triangle with coordinates (4, 3) is the starting point. The coordinate (17, 16) is the end point.

**Figure 9:**
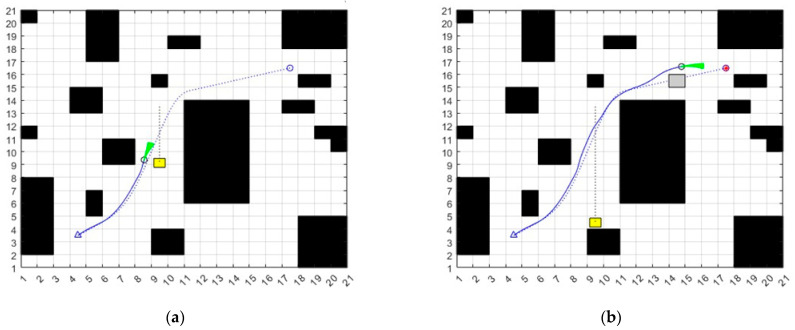
(**a**) Plan path in dynamic obstacle map, (**b**) Plan path in mixed obstacle map.

In the map, the black squares are obstacles. The triangle is the starting point, the circle is the goal point. The circle with the green arc is the mobile robot. The yellow square is the moving obstacle, the gray square is the temporary static obstacle. The blue dashed line is the global path, and the blue solid line is the final generated path. To verify the global path planning and the ability to avoid local dynamic obstacles of the improved A* fusion DWA algorithm, a 21 m × 21 m raster map is selected for the experiment. A movable obstacle is set in the path of the robot. The simulation experiment results are shown in Figure 9.

**Figure 10:**
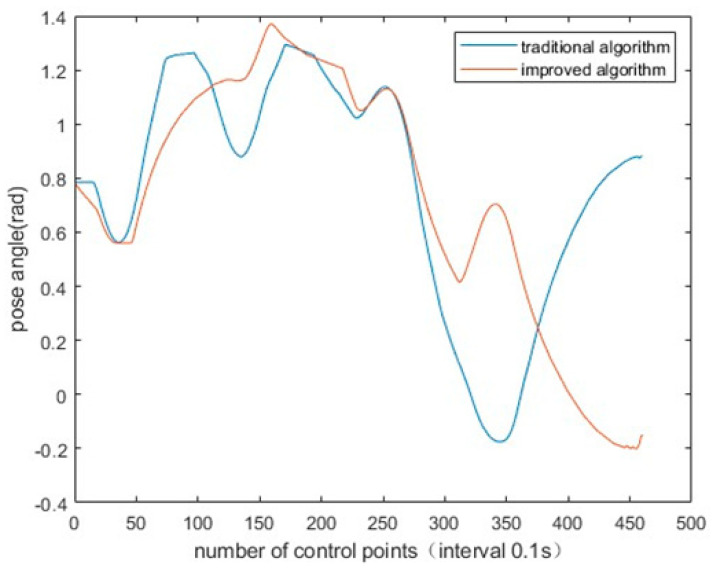
Improved algorithm and traditional algorithm for AGV pose.

**Figure 11:**
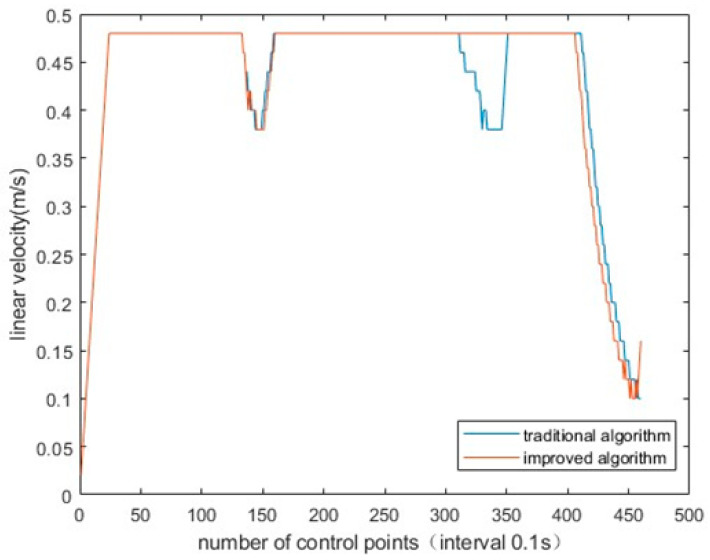
Improved algorithm and traditional algorithm for AGV linear velocity.

**Figure 12:**
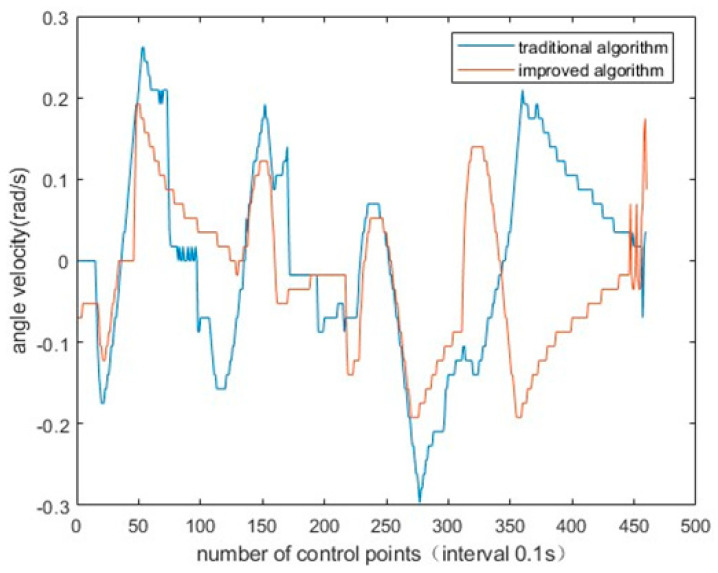
Improved algorithm and traditional algorithm for AGV angle velocity.

To verify the improved evaluation function of DWA algorithm, the linear velocity, angular velocity and pose of AGV were, respectively, generated by traditional DWA algorithm and improved DWA algorithm. It can be obviously found that the linear velocity fluctuation of AGV is reduced, the angular velocity fluctuation is smaller and the posture is better. The simulation experiment results are shown in Figure 10, Figure 11 and Figure 12.

**Figure 13:**
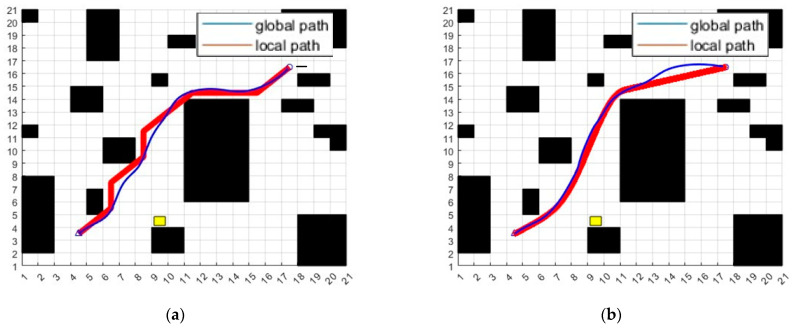
(**a**) Path of the hybrid algorithm of traditional A* algorithm and traditional DWA algorithm. (**b**) Path of the fusion algorithm that combines kinematical constraint A* algorithm and follows DWA algorithm.

To verify the fitting of local path to global path, the linear velocity, the paths generated by the hybrid algorithm of traditional A* algorithm and traditional DWA algorithm and the paths generated by the fusion algorithm that combines the kinematical constraint A* algorithm and the following DWA algorithm are generated, respectively. The path in red is the global path. The blue path is the local path. The yellow grid at coordinate (9, 4) is the termination position of the movable obstacle. There is a temporary stationary obstacle at coordinate (14, 15). It can be obviously found that the path of the fusion algorithm is better. The simulation experiment results are shown in Figure 13.

The running results of Table 2, Table 3 and Table 4 show that the global path length has been reduced 3.571% by improved A* algorithm. The time of the local path has been reduced 50% by an improved DWA algorithm. Compared with the fusion algorithm of traditional A* algorithm and traditional DWA algorithm, the hybrid algorithm proposed in this paper can plan an optimal path, which can reduce the number of turns of the final path by 25%.

## 5. Conclusions

This paper introduces a fusion algorithm that combines the kinematical constraint A* algorithm and the following dynamic window approach. Firstly, optimize the search strategy for child nodes. Secondly, improve the heuristic function of the A* algorithm. Thirdly, remove redundant points of path. Fourthly, create a B spline curve smooth path. Finally, improve the heuristic function of the DWA algorithm. Compared with the fusion algorithm of traditional A* algorithm and traditional DWA algorithm, the fusion algorithm reduces length of path by 3.6%, time of path by 6.7% and the number of turns of the final path by 25%. The local path is closer to the global path. AGV has lower linear velocity, smaller angular velocity and better pose angle. AGV can avoid dynamic obstacles.

The fusion algorithm proposed in this paper is better than other traditional algorithms, but it still has the following deficiencies:

(1) In fusion algorithm, the kinematic characteristics of the mobile robot are considered. The energy efficiency of the mobile robot is not considered. This may not save energy on mobile robots;
(2) The fusion algorithm in this paper does not integrate environmental awareness. It only carries out path planning on the established map. There is a need to integrate efficient perception methods.

However, there are still a lot of future studies required in the field of robot path planning:

(1) The fusion algorithm is applied to a real mobile robot. Verify the feasibility of fusion algorithm in a real mobile robot. The fusion algorithm is tested on different mobile robots. Verify the compatibility of the fusion algorithm. Test the fusion algorithm in different environments. Some coefficients in the fusion algorithm are adjusted for different environments;
(2) Coordinate and control robot clusters to prevent conflicts. Multi-machine collaboration can expand the working radius of the mobile robots. Autonomously assign multiple tasks. Multi-task assignment can make the mobile robots work more efficiently. Research is a hot topic.

## Figures and Tables

**Table 1:** Child node search direction strategy.

Θ	Keep 5 Directions	Abandon Direction
[337.5°, 360°) ∪ [0°, 22.5°)	000T, 045T, 090T, 270T, 315T	135T, 180T, 225T
[22.5°, 67.5°)	000T, 045T, 090T, 135T, 315T	180T, 225T, 270T
[67.5°, 112.5°)	000T, 045T, 090T, 135T, 180T	225T, 270T, 315T
[112.5°, 157.5°)	045T, 090T, 135T, 180T, 225T	270T, 315T, 000T
[157.5°, 202.5°)	090T, 135T, 180T, 225T, 270T	000T, 045T, 315T
[202.5°, 247.5°)	135T, 180T, 225T, 270T, 315T	000T, 045T, 090T
[247.5°, 292.5°)	180T, 225T, 270T, 315T, 000T	045T, 090T, 135T
[292.5°, 337.5°)	225T, 270T, 315T, 000T, 045T	090T, 135T, 180T

**Table 2:** Comparison of different algorithms’ performances.

Map/m^2^	Algorithm	Path Length (m)	Number of Turns	Path Time (s)
21 × 21	Traditional A* + DWA	35.39	13	125
	Improved A* + DWA	31.68	11	120
	Traditional A* + Traditional DWA	20.72	4	64.5
	Improved A* + Improved DWA	19.98	3	60.2

**Table 3:** Comparison of algorithm performance in different maps.

Map/m^2^	Obstacles	Path Length (m)	Number of Turns	Path Time (s)
21 × 21	dynamic obstacle	19.93	3	59.6
	mixed obstacles	20.2	4	60.2

**Table 4:** Comparison of algorithms in this paper.

Algorithm	Global Optimality	Smooth Path	Local Optimality	Deceleration Obstacle Avoidance	Dynamic Obstacle Avoidance
Tradition A*	T	F	F	F	F
Improved A*	T	T	F	F	F
Hybrid Algorithm	T	T	T	T	T

## Data Availability

Not applicable.

## Appendix A

(A1)
$$P(t)=\sum_{i=0}^{3}P_{i}B_{i,3}(t)$$

(A2)
$$\left\{\begin{array}{c} B_{0,3}(t)=\frac{1}{6}(-t^{3}+3t^{2}-3t+1)\\ B_{1,3}(t)=\frac{1}{6}(3t^{3}-6t^{2}+4)\\ B_{2,3}(t)=\frac{1}{6}(-3t^{3}+3t^{2}+3t+1)\\ B_{3,3}(t)=\frac{1}{6}t^{3} \end{array}\right.t\in [0,1]$$

(A3)
$$P(t)=\frac{1}{6}[t^{3}(-P_{0}+3P_{1}-3P_{2}+P_{3})+t^{2}(3P_{0}-6P_{1}+3P_{2})+t^{}(-3P_{0}+3P_{2})+(P_{0}+4P_{1}+P_{2})]$$
